# Validation of a Patient-Specific Musculoskeletal Model for Lumbar Load Estimation Generated by an Automated Pipeline From Whole Body CT

**DOI:** 10.3389/fbioe.2022.862804

**Published:** 2022-07-11

**Authors:** Tanja Lerchl, Malek El Husseini, Amirhossein Bayat, Anjany Sekuboyina, Luis Hermann, Kati Nispel, Thomas Baum, Maximilian T. Löffler, Veit Senner, Jan S. Kirschke

**Affiliations:** ^1^ Associate Professorship of Sport Equipment and Sport Materials, School of Engineering and Design, Technical University of Munich, Munich, Germany; ^2^ Department of Diagnostic and Interventional Neuroradiology, School of Medicine, Klinikum rechts der Isar, Technical University of Munich, Munich, Germany; ^3^ Department of Informatics, Technical University of Munich, Munich, Germany; ^4^ Department of Diagnostic and Interventional Radiology, University Medical Center Freiburg, Freiburg im Breisgau, Germany

**Keywords:** musculoskeletal multibody dynamics, spinal biomechanics, patient-specific, lumbar alignment, automated model generation, spinal loading, muscle force computation, chronic back pain

## Abstract

**Background:** Chronic back pain is a major health problem worldwide. Although its causes can be diverse, biomechanical factors leading to spinal degeneration are considered a central issue. Numerical biomechanical models can identify critical factors and, thus, help predict impending spinal degeneration. However, spinal biomechanics are subject to significant interindividual variations. Therefore, in order to achieve meaningful findings on potential pathologies, predictive models have to take into account individual characteristics. To make these highly individualized models suitable for systematic studies on spinal biomechanics and clinical practice, the automation of data processing and modeling itself is inevitable. The purpose of this study was to validate an automatically generated patient-specific musculoskeletal model of the spine simulating static loading tasks.

**Methods:** CT imaging data from two patients with non-degenerative spines were processed using an automated deep learning-based segmentation pipeline. In a semi-automated process with minimal user interaction, we generated patient-specific musculoskeletal models and simulated various static loading tasks. To validate the model, calculated vertebral loadings of the lumbar spine and muscle forces were compared with *in vivo* data from the literature. Finally, results from both models were compared to assess the potential of our process for interindividual analysis.

**Results:** Calculated vertebral loads and muscle activation overall stood in close correlation with data from the literature. Compression forces normalized to upright standing deviated by a maximum of 16% for flexion and 33% for lifting tasks. Interindividual comparison of compression, as well as lateral and anterior–posterior shear forces, could be linked plausibly to individual spinal alignment and bodyweight.

**Conclusion:** We developed a method to generate patient-specific musculoskeletal models of the lumbar spine. The models were able to calculate loads of the lumbar spine for static activities with respect to individual biomechanical properties, such as spinal alignment, bodyweight distribution, and ligament and muscle insertion points. The process is automated to a large extent, which makes it suitable for systematic investigation of spinal biomechanics in large datasets.

## 1 Introduction

Chronic back pain is considered a major burden for patients and healthcare systems worldwide. Though general risk factors, such as occupation, obesity, or anthropometric parameters, could be identified in the past years (Murtezani et al., 2011), specification of individual indicators for the prediction of symptoms and chronicity is challenging. The invasive character of *in vivo* measurement via intradiscal pressure sensors (Sato et al., 1999; Wilke et al., 2001) or instrumented vertebral implants (Rohlmann et al., 2008; Dreischarf et al., 2016) makes these methods unsuitable for clinical analysis. Computational biomechanical models can provide a valuable alternative when it comes to the estimation of spinal loads. However, biomechanics of the human spine are subject to a variety of influencing factors, such as spinal alignment, body weight distribution, the function of muscles, degeneration of connective tissues, and other preconditions of the musculoskeletal system. Due to the highly individual character of these factors, as many of them as possible should be considered during the modeling process to generate meaningful biomechanical models of the spine. The assessment of relevance to accounting for biological variation in biomedical engineering regarding modeling was the subject of several studies (Cook et al., 2014; Bruno et al., 2017; Akhavanfar et al., 2018; Iyer et al., 2018).

Biomechanical models have been widely used to gain insights into healthy and pathological biomechanics of the spine. While finite element models exist that account for individual characteristics (Akhavanfar et al., 2018; El Ouaaid et al., 2016; Eskandari et al., 2019; Ghezelbash et al., 2016; Little and Adam, 2015; Naserkhaki et al., 2016; Périé et al., 2002; Vergari et al., 2016), multibody models are predominantly generic or focus on specific pathologies such as adolescent idiopathic scoliosis (Jalalian et al., 2017; Petit et al., 2004). The neglect or only limited consideration of interindividual variation makes these models poorly suited for a detailed subject-specific analysis. In recent years, several such models were published (Delp et al., 2007; Christophy et al., 2012; Bruno et al., 2015; Actis et al., 2018; Favier et al., 2021; Bassani et al., 2019; de Zee et al., 2007; Han et al., 2012; Kim and Zhang, 2017; Liu et al., 2019). These generic models are often based on average anthropometric data (Bassani et al., 2019; de Zee et al., 2007; Han et al., 2012; Kim and Zhang, 2017; Liu et al., 2019) or detailed models based on cadaver studies (Bayoglu et al., 2017a; b, 2019). The necessary input to create accurate, individualized models can be provided by imaging data (Senteler et al., 2014; Dao et al., 2015; Dao et al., 2015; Hajihosseinali et al., 2015; Bruno et al., 2017; Favier et al., 2021). A study recently published by Fasser et al. introduced a pipeline for the generation of semi-individualized multi-body models of the spine based on manually annotated EOS imaging data (Fasser et al., 2021). In general, individualization of biomechanical models usually involves a time-consuming, manual or semi-automated process, which requires expert knowledge and therefore, makes it poorly suited for clinical applications.

On the way to integration of patient-specific numerical models in clinical practice, Zadpoor et al. identified two key parameters: accuracy and cost-effectiveness (Zadpoor and Weinans, 2015). While the aspect of accuracy can be covered by using imaging data (Blemker et al., 2007), the aspect of cost-effectiveness should be addressed by automating involved processes to a large extent. In 2021, Cina et al. published a deep learning process to identify landmarks for vertebral corners from radiographs (Cina et al., 2021). To this date, automated approaches for modeling from medical imaging are rare in the literature. In 2021, Caprara et al. introduced the first automated pipeline for the generation of patient-specific finite element models of the functional spine unit using a combination of deep learning, statistical, and FE methods on 3D CT scans (Caprara et al., 2021). To the best of our knowledge, a similar approach for multi-body modeling does not exist in the literature.

We established the first framework for a fully automated pipeline to derive individual biomechanical models from imaging data for the estimation of spinal loads to determine functional anthropometric parameters. The objective of this study is the validation of a musculoskeletal model of the torso with subject-specific spinal geometries and soft tissue distribution.

## 2 Methods

Input data for the automated modeling process were derived from a deep learning-based pipeline for automated vertebrae segmentation and extraction of spinal characteristics from CT scans. We incorporated a detailed muscle architecture for the lumbar region, simulated various static activities, and compared estimated muscle forces and vertebral loading with *in vivo* data from the literature. Finally, an interindividual analysis of two models derived from two datasets served as proof of concept for the potential of our process to systematically investigate individual spinal loading.

### 2.1 Automated Extraction of Spinal Geometries and Points of Interest

**FIGURE 1:**
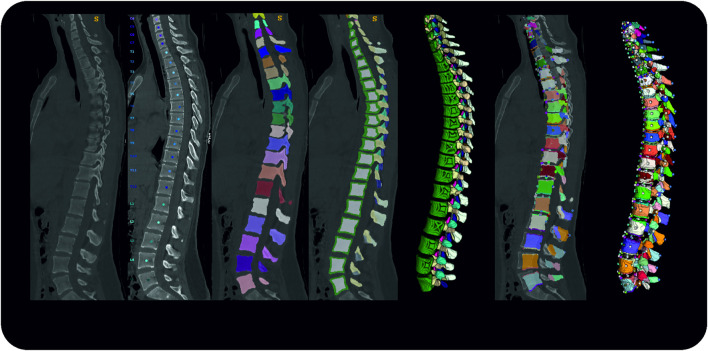
Pipeline Overview from left to right; original data, vertebrae identification; vertebrae segmentation; subregion segmentation (cross-section and 3D rendering); re-alignment in craniocaudal direction and calculation of points of interest; 3D rendering of the final dataset.

The processing and extraction of patient data described in the following section were executed from asynchronously phantom-based calibrated CT image data (Kaesmacher et al., 2017). We labeled and segmented vertebrae using an automated deep learning-based process for vertebrae segmentation, which is described in detail elsewhere (Sekuboyina et al., 2020). In brief, three artificial neural networks (ANNs) are used to 1) detect the spine, 2) identify and label each vertebra as well as 3) segment each vertebra based on the label. The latter two steps were reviewed by a radiologist and could potentially be corrected. For each vertebra, centroids, as well as segmentation masks for eleven subregions, were generated using a fourth ANN: vertebral body (further divided into the cortex and the trabecular compartment), vertebral arch, spinous process, as well as transverse processes. Before calculating necessary points of interest, the centroids of the first thoracic and last lumbar vertebra were aligned vertically to account for posture differences between supine from CT scans and upright position. Thereafter, these data were used to calculate relevant points for muscle and ligament attachments. Figure 1 shows the overall process.

**FIGURE 2:**
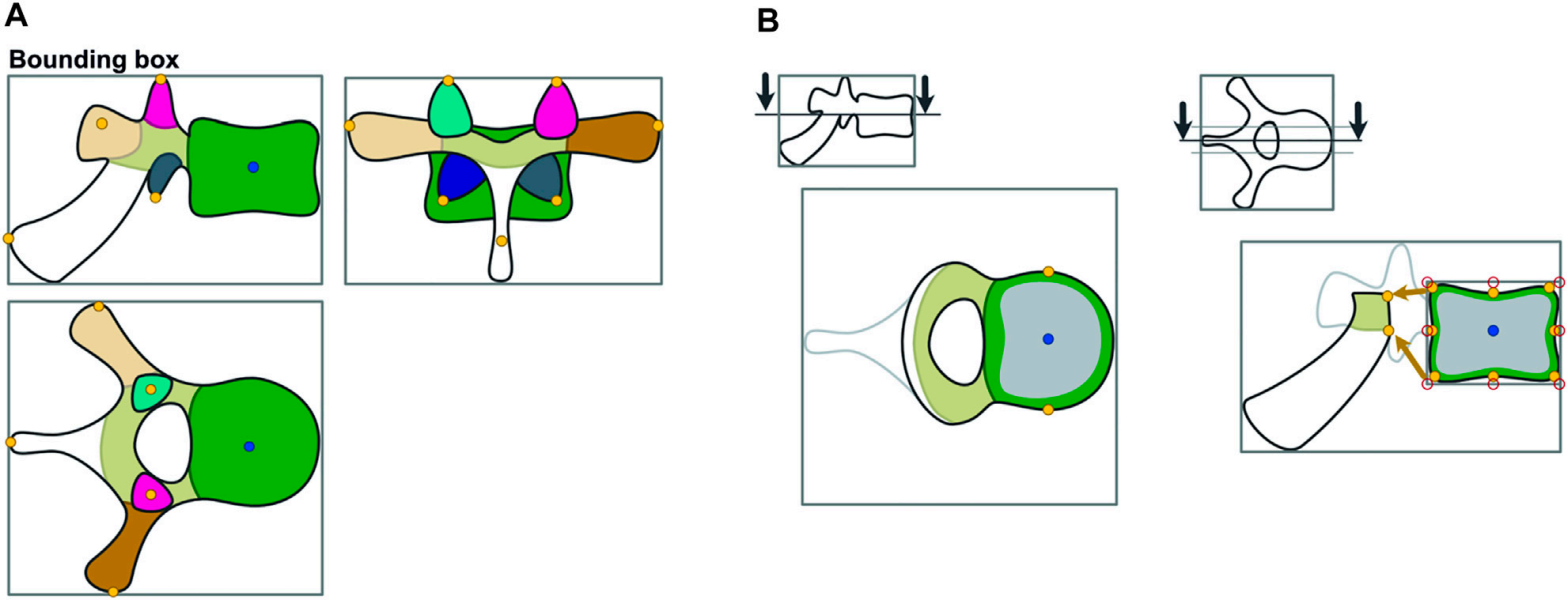
Calculation of points of interest. The vertebra is divided in subregions, which are used further to identify landmarks based on geometrical extreme values **(A)**. Horizontal and auxiliary planes are inserted to identify points of interest of the vertebral body via a projection of the border points of a bounding box on the respective subregion **(B)**.

Subsequently, we defined points of interest for each individual vertebra. Therefore, the algorithm iterated over each vertebra, creating bounding boxes based on its binary segmentations. Corresponding to those bounding boxes, individual subregion segmentations were used to define landmarks for muscle and ligament attachment points by geometrical extreme values as shown in Figure 2A. Thus, depending on the subregion, the most posterior, inferior, superior, or lateral point on the surface was determined by its minimal and maximal coordinate values along the corresponding spatial axis. Based on centroid positions, auxiliary sagittal and horizontal planes were set through the vertebral body to extract its attachment points (Figure 2B). In the horizontal sectional plane, the most lateral points on each side of the vertebral body were extracted. In the sagittal sectional plane, a rectangle is fitted around the subregion of the vertebral body. The corner and center points of the rectangle border were then projected onto the surface by the shortest distance. Using a similar function, attachment points on the vertebral arch were determined via the minimal distance between the anterior border of its sectional plane and posterior points in the vertebral body. The plane was then shifted right and left and the process was repeated.

**FIGURE 3:**
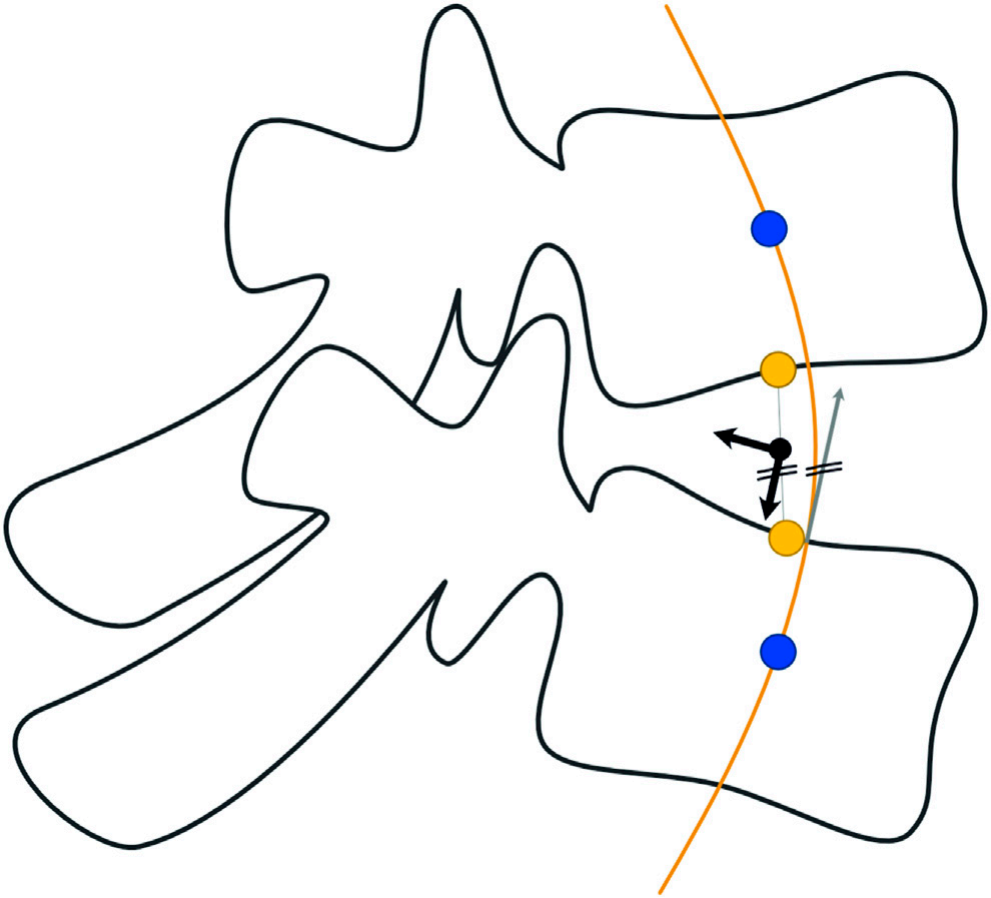
Determination of intervertebral joint location and orientation. The position of the joint is assumed to be the midpoint of the straight line connecting the central points of the endplates. Orientation of the marker is defined via the derivative of intersection of the spline interpolation of vertebral centroids.

We assumed the location of the intervertebral joint to be the midpoint of the straight line connecting the central points of the lower and upper endplates of the two vertebrae representing one motion segment (Figure 3). We used a spline interpolation of all vertebral body centroids to define intervertebral joint orientation by calculating the spline derivative at the intersection with the upper endplate of the inferior vertebra.

To account for individual torso weight distribution, a segmentation mask for lung, fat, and muscle/organ tissue was created based on typical CT intensity ranges. Subsequently, the segmented tissue was assigned to the nearest vertebra depending on its minimal anterior–posterior distance. Thus, the torso weight was subdivided into segments for each vertebral level. For each segment, the algorithm calculated its center of mass and total weight corresponding to its individual tissue distribution. We assume to have an average density of 0.25 g/cm^3^ for the lung, 0.96 g/cm^3^ for fat, and 1.06 g/cm^3^ for the remaining soft tissues (Pearsall et al., 1996; Akhavanfar et al., 2018).

### 2.2 Individualized Musculoskeletal Model of the Thoracolumbar Spine

**FIGURE 4:**
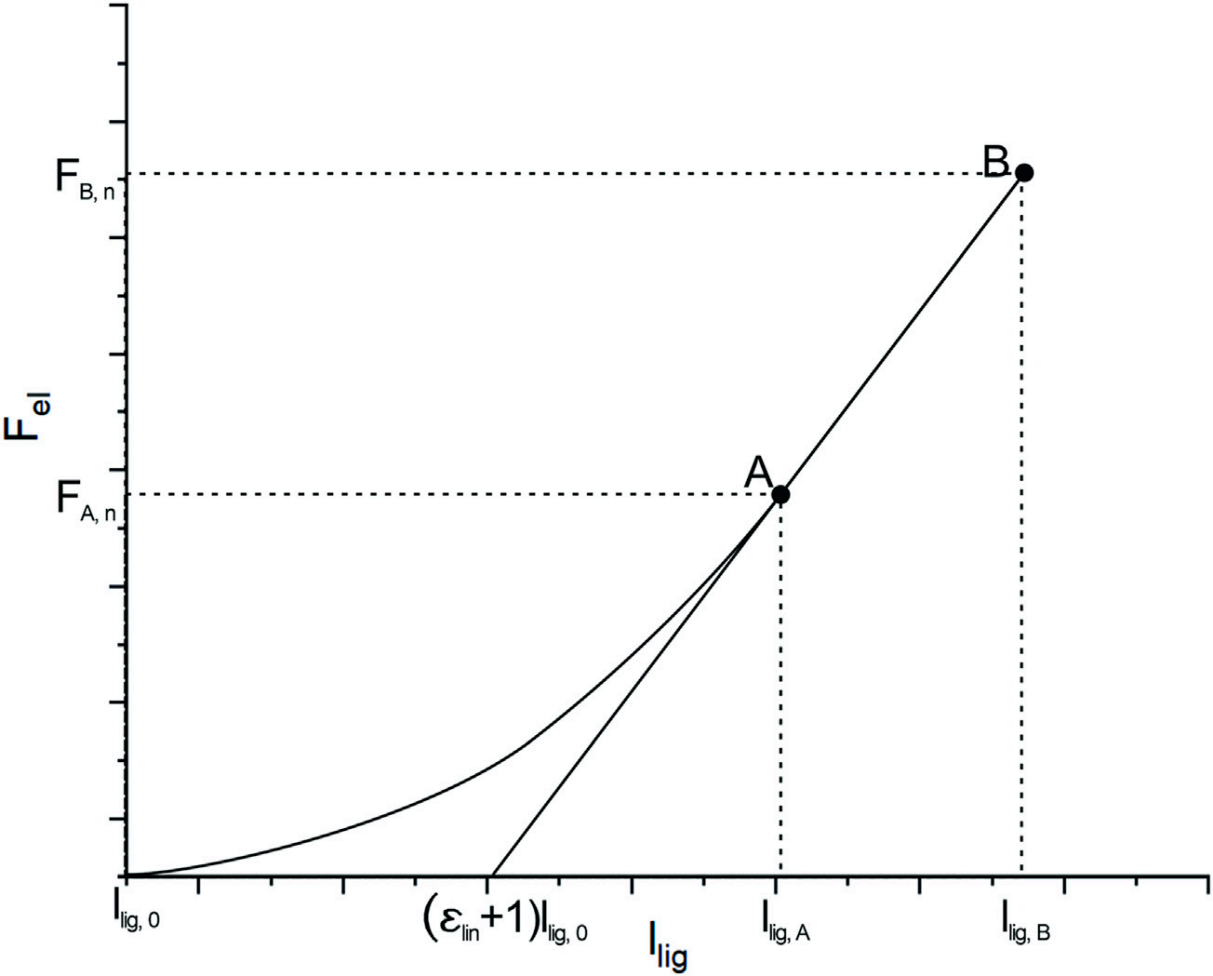
Typical force–length curve to describe the mechanical behavior of ligaments. Transition from nonlinear to linear regions are defined by l_A_ and F_A_ and F_B_ and l_B_ marks the maximum force and elongation before failure occurs.

The automated modeling of the thoracolumbar spine with generic bodies of the head–neck complex, ribcage, simplified upper extremities, and the pelvic-sacral region is carried out using the multibody simulation software SIMPACK (Dassault systèmes, France). The thoracolumbar spine includes individual information on vertebrae T1-L5, insertion points for muscles and ligaments, spinal alignment, as well as paraspinal soft tissue distribution as described in the previous chapter. Bodies for the head–neck system, ribcage, sacrum, and pelvis are individually scaled according to Winter (2005) and equipped with relevant points for muscle insertion and integrated into the model. Neglecting facet joints and intraabdominal pressure, lumbar intervertebral joints are modeled as actuated spherical joints to ensure necessary stability. The thoracic spine, head–neck, and ribcage are modeled as one rigid body, and segment masses for soft tissue are rigidly fixed to each vertebra according to the calculated centers of mass. Segment masses relative to overall torso mass calculated from the CTs (Winter, 2005) were assigned to the bodies for head–neck and simplified arms. The masses of bony structures were calculated assuming a density of 1.5 g/cm^3^ (Pearsall et al., 1996; Akhavanfar et al., 2018). Intervertebral discs, as well as ligaments, are modeled as nonlinear, viscoelastic force elements. Occurring moments in the intervertebral discs are characterized by a nonlinear load–deformation relationship (Rupp et al., 2015; White, 2022). Specific data on stiffness and neutral zones of the intervertebral discs were taken from White (2022). The model includes anterior and posterior longitudinal ligament (ALL and PLL), flavum ligament (LF), interspinal ligament (ISL), and supraspinal ligament (SSL). The characteristic force–length curve for the elastic behavior of ligaments (Figure 4) shows a nonlinear toe region in the region of small deformations (A), followed by a linear elastic region before the final failure of the ligament (B). Inspired by the study cited herein(Rupp et al., 2015), their nonlinear force–length characteristics are described in Eq. 1.

$$F_{el}\left(l_{\mathrm{lig}}\right)=\left\{\begin{array}{cc} 0,\; & \text{for}\;l_{\mathrm{lig}}\leq l_{\mathrm{lig,0}}\\ K_{nl}{\left(l_{\mathrm{lig}}-l_{\mathrm{lig,0}}\right)}^{exp_{\mathrm{nll}}},\; & \text{for}\;l_{\mathrm{lig}}\leq l_{\mathrm{lig,A}}\\ F_{A,n}+K_{\mathrm{lin}}\left(l_{\mathrm{lig}}-l_{\mathrm{lig,A}}\right),\; & \text{for}\;l_{\mathrm{lig}}>l_{\mathrm{lig,A}} \end{array}\right.$$ (1)

with

**Table:** 

*l*_*lig,0*_ = (1 − *ϵ*_*pre*_)*l*_*neut*_	, where l_neut_ is the individually measured length for each ligament segment in neutral position and *ϵ*_pre_ is the individual pre-strain
$F_{A/B,n}=\frac{F_{A/B}}{n}$	, where n is the number of parallel components for each ligament and F_A_ is the ligament force at point A (same for B)
*l*_*lig,A∕B*_ = (1 + *ϵ*_*A*/*B*_)*l*_*lig,0*_	, where l_A_ is the length at point A and *ϵ*_A_ (same for B)
$K_{\mathrm{lin}}=\frac{F_{B,n}-F_{A,n}}{l_{\mathrm{lig,B}}-l_{\mathrm{lig,A}}}$	, where K_lin_ is the elastic stiffness in the linear region
$\epsilon_{\mathrm{lin}}=\frac{F_{A,n}}{K_{\mathrm{lin}}l_{\mathrm{lig,0}}}$	, where *ϵ*_lin_ is the strain at the intersection of the applied tangent from the linear region with the abscissa
$exp_{\mathrm{nll}}=\frac{\epsilon_{A}}{\epsilon_{\mathrm{lin}}}$	, where exp_nll_ defines the order of non-linearity of the toe region
$K_{nl}=\frac{F_{A,n}}{{\left(\epsilon_{A}l_{\mathrm{lig,0}}\right)}^{exp_{\mathrm{nll}}}}$	, where K _nl_ is the individual factor that defines stretch/compression of the toe-region

Individual lengths for ligament segments are measured directly in the model in neutral position, which is what we considered upright standing. Values for initial ligament lengths were calculated considering values for pre-strain from the studies cited herein (Nachemson and Evans, 1968; Aspden, 1992; Robertson et al., 2013). The main difference to the mechanical law provided by Rupp et al. is that we take relative strain values for l_lig, A_ and l_lig, B_ instead of absolute elongations. Therefore, we guarantee uniform preloads within one ligamentous structure for the neutral position. Parameters for strains *ϵ*_A_,*ϵ*_B_, and Forces F _A_ and F _B_ at points A and B, are taken from the study referred herein (Chazal et al., 1985).

### 2.3 Muscle Force Calculation

**FIGURE 5:**
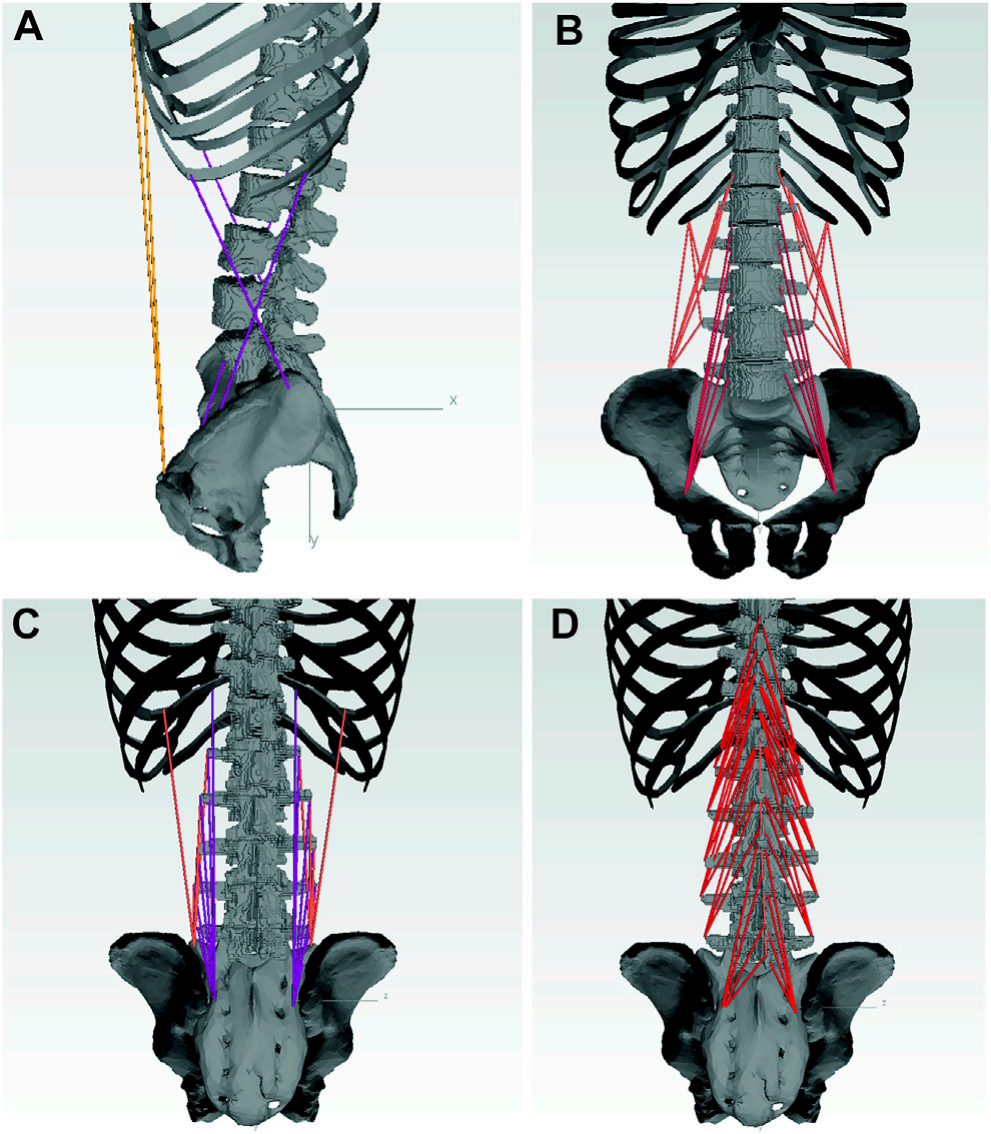
Musclegroups included in the model with: RA, EO, IO **(A)**; QL, PM **(B)**; LL, IL **(C)**; MF, IS **(D)**.

Nine muscle groups of the lower back and abdomen are included in the model as 103 point-to-point actuators: rectus abdominis (RA), internal obliques (IO), external obliques (EO), psoas major (PM), quadratus lumborum (QL), multifidus (MF), longissimus thoracis pars lumborum (LL), iliocostalis lumborum (IL) and the interspinales lumborum (IS) (Figure 5). Globally acting muscles RA, IO, and EO, as well as those muscle fascicles of LL, QL, and IL attached to the ribcage, are simplified each to one actuator per side. Muscles acting locally on the lumbar spine are modeled in detail based on attachment points taken from a cadaver study (Bayoglu et al., 2017a). Muscle fascicles attached to the same subregion were combined and physiological cross-sectional areas (PCSA) based on Christophy et al. (2012) were assigned to respective fascicles.

The MBS model calculates necessary joint moments M to hold the imposed static positions, which are transferred to Matlab via a SIMULINK model, where muscle forces are calculated using a static optimization approach (Gagnon et al., 2001). In order to increase the chances of finding a global optimum, we used the globalsearch solver (Global Optimization Toolbox, Matlab 2020b) to solve the following optimization problem:

$$Minimize\left(CostFunktion=\overset{n}{\underset{i=1}{\sum }}{\left(\frac{F_{i}}{PCSA_{i}}\right)}^{3}\right)$$ (2)

subject to equality constraints

$$c_{eq}=\left(\begin{array}{c} M_{x,1}\\ \ldots \\ M_{z,i} \end{array}\right)=0$$ (3)

and bound constraints

$$0\leq F_{i}\leq \sigma_{max}{PCSA}_{i}$$ (4)

Focusing on vertebral loading in the sagittal and frontal plane, equality constraints consider respective moments (x for frontal, z for sagittal) occurring in each lumbar intervertebral joint. Only active forces are taken into account neglecting the passive elastic behavior of muscular tissues. To guarantee compliance with the equilibrium conditions for all load cases, maximal muscle stress (MMS) was first set to 0.6 MPa, (Arjmand et al., 2009), and then to 1 MPa (Bruno et al., 2015; Beaucage-Gauvreau et al., 2019; Favier et al., 2021).

### 2.4 Individual Characteristics of Selected Subjects

**FIGURE 6:**
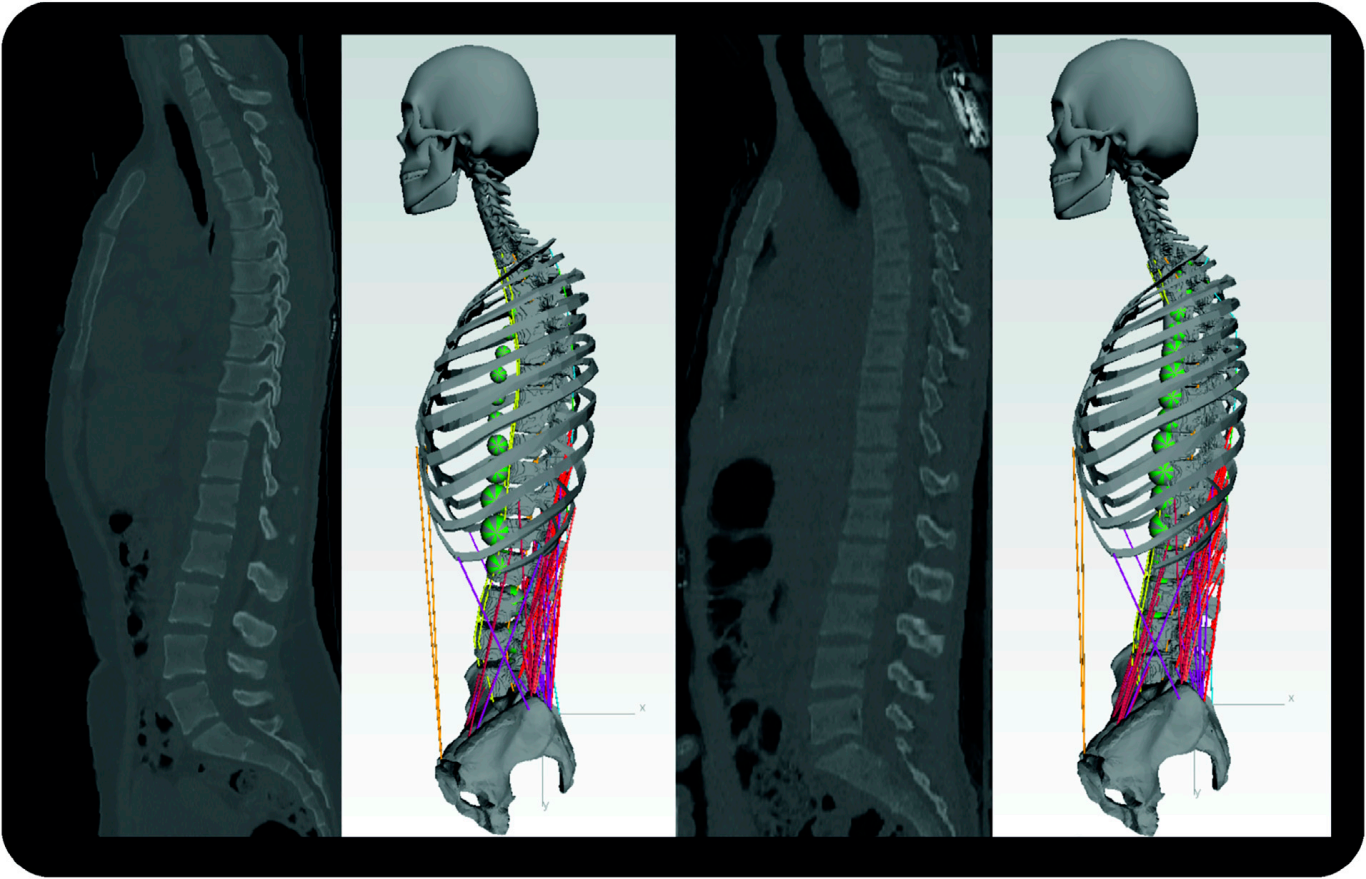
Sagittal CT images of subject one (left) and subject two with respective MBS models. Segment masses for soft tissues are visualized by the green spheres. For the sake of clarity, the dummy bodies for the arms are not shown here.

For model validation, we built models based on two nondegenerative spine datasets (Figure 6). We selected datasets of two young patients (1 M, 1 F) with anthropometric data as comparable as possible to the subjects in comparative studies (Wilke et al., 2001; Takahashi et al., 2006; Rohlmann et al., 2008).

Anthropometric data, such as body height and weight, was calculated in reference to individual spine height and torso weight from CT data according to the study cited herein Winter (2005). To characterize individual spinal alignment, we measured kyphosis and lordosis angles for T1-T12, and L1-S1 in the sagittal plane, as well as Cobb angles for C7-T12, in the frontal plane and these are summarized in Table 1.

**TABLE 1:** Anthropometric data and parameters on the individual spinal alignment of selected subjects.

	Anthropometric Data	Lumbar lordosis	Thoracic kyphosis	Thoracic skoliosis
Subject 1	F	29°	31°	18°
	1.76 m	—	—	right-convex
	65 kg	—	—	—
Subject 2	M	11°	27°	—
	1.73 m	—	—	—
	86 kg	—	—	—

### 2.5 Model Validation

We evaluated predicted muscle forces and spinal loading for various activities. The load cases were selected based on previous studies on the measurement of intradiscal pressure and muscle activation (Wilke et al., 2001; Rohlmann et al., 2008). Table 2 summarizes all investigated load cases.

**TABLE 2:** Load cases taken from *in vivo* studies used for validation of the model. Spinal loading was measured using intradiscal pressure (IDP) sensors (Wilke et al., 2001; Takahashi et al., 2006) or instrumented vertebral implants (Rohlmann et al., 2008).

Load cases	Subject (M)	Study type	Measured	References
Standing	1	*In vivo*	IDP L4/L5	Wilke et al. (2001)
Standing with 20 kg 20 cm from chest	—	—	—	—
Standing with 20 kg 55 cm from chest	—	—	—	—
Standing	2	*In vivo*	L1 Implant Load	Rohlmann et al. (2008)
30 deg Flexion	—	—	—	—
Elevate both arms	—	—	—	—
Standing (w/o weight and 10 kg)	3	*In vivo*	IDP L4/L5	Takahashi et al. (2006)
10 deg Flexion (w/o weight and 10 kg)	—	—	—	—
20 deg Flexion (w/o weight and 10 kg)	—	—	—	—
30 deg Flexion (w/o weight and 10 kg)	—	—	—	—

Since the subjects from *in vivo* studies were all men (Table 2), we only used the model based on the dataset of the male for validation. Prior to the simulation of dedicated load cases, we used an optimization routine to determine the optimal position for upright standing. Assuming the optimal position to be energy efficient, joint angles of the lumbosacral spine (T12—Sacrum) were optimized, subject to minimization of occurring joint moments in the sagittal plane. We adopted determined joint angles as the starting posture for a neutral stance in the further course.

Applied flexion was assumed to be 40% sacral rotation and 60% lumbar flexion (Liu et al., 2018). According to the studies referred herein (Wong et al., 2006; Christophy et al., 2012), lumbar flexion was distributed 25.5% for L1/L2, 23.1% for L2/L3, 20.4% for L3/L4, 18.5% for L4/L5, and 12.5% for L5/S1.

Ligament modeling was evaluated based on their stress states during each load case with a focus on whether they were within a physiological range or whether failure could already be expected. Calculated lumbar loads were compared to respective vertebral load measurements and predicted muscle forces were evaluated in the context of measured EMG signals from experimental studies.

### 2.6 Potential for Systematic Analysis of Spinal Loads—Proof of Concept

To determine the potential of our pipeline regarding the systematic investigation of individual spinal loads, we compared generated results for both patients considering their individual spinal alignment. For this, compensation angles for an upright position, as well as estimated muscle forces and spinal loads, were compared, put into context with the identified curvature of each subject in the frontal and sagittal plane, and analyzed for plausibility.

## 3 Results

### 3.1 Ligament Forces

**FIGURE 7:**
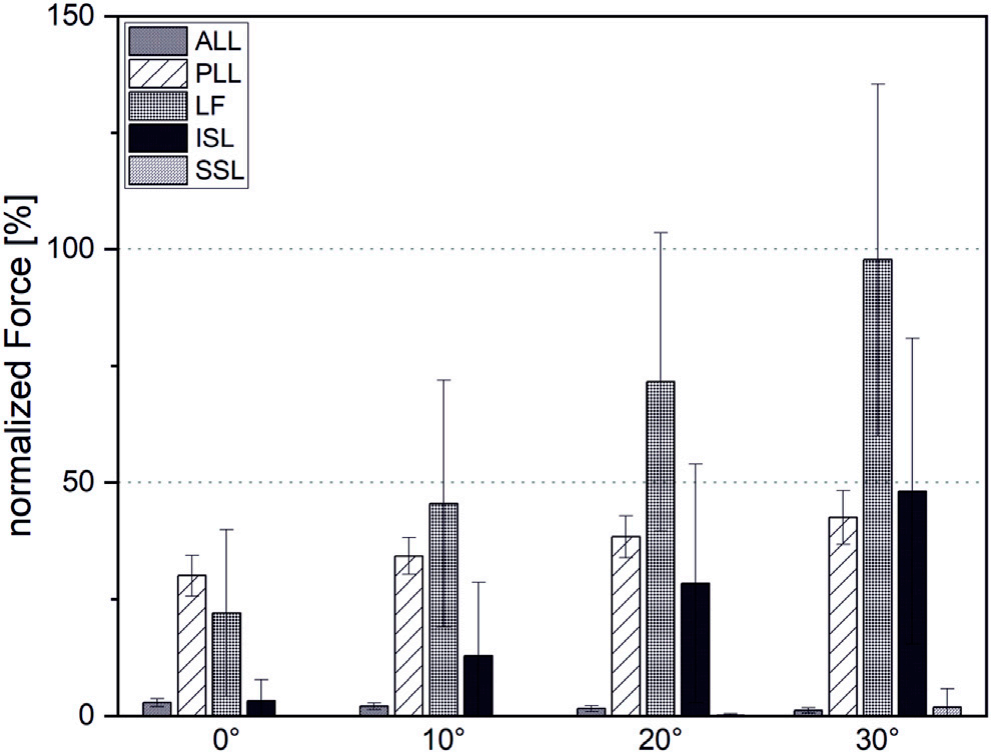
Ligament forces normalized to maximal force at the end of the linear region before failure.

For initial simulations with LF pre-strain taken from the study referred herein (Nachemson and Evans, 1968), the occurring LF forces exceeded physiological maximum forces even for low-intensity flexions ( < 10°). We reduced pre-strain from 10% to 5%, which still lies within the standard deviation of experimentally determined values (Nachemson and Evans, 1968) (Figure 7). Mean normalized forces were within a physiological range for all ligaments during investigated loading tasks. However, despite adjusted pre-strain, the average LF force reaches close to 100% with standard deviations of up to 37% during 30°flexion. The remaining ligament showed low (ALL, PLL, SSL) to moderate (PLL, ISL) loading, with a less than 2% and 50%, respectively.

### 3.2 Muscle Force Estimation

**FIGURE 8:**
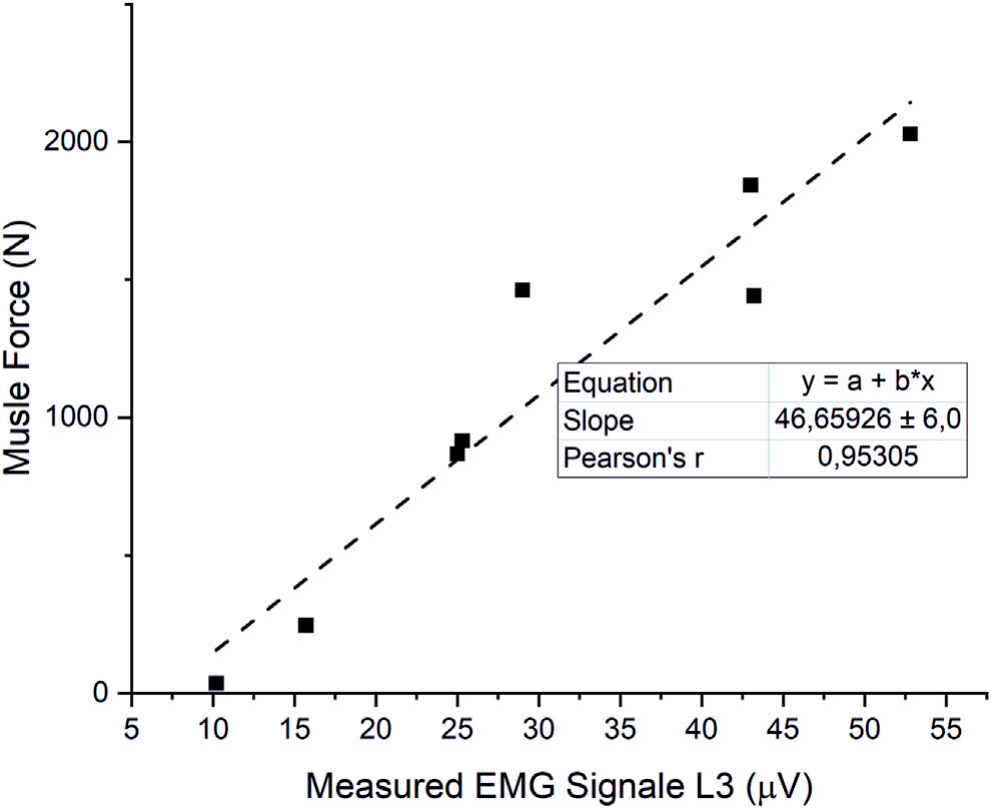
Correlation of estimated ES forces and measured EMG signals from (Takahashi et al., 2006).

The estimated muscle forces of the erector spinae correlated closely (r = 0.95) with measured EMG-signals from Takahashi et al. (2006) (Figure 8). Initial simulations with a detailed muscular architecture according to Bayoglu et al. (2017a), under consideration of physiological MMS 60 N/cm^2^, could not satisfy equilibrium conditions for all models, even for moderately intense activities, such as 30° flexion. Even increasing the MMS to 1 MPa was not sufficient to reliably satisfy the equilibrium conditions for all cases, though this mainly affected high-intensity cases, such as extensive flexion with additional weight. Therefore, we adapted muscle properties according to a validated musculoskeletal model from literature (Christophy et al., 2012). However, Christophy’s model includes no muscle fascicles for the IS, nor those fascicles of the MF attached to the thoracic spine, which we added and in order to guarantee compliance with the equilibrium conditions, equipped with PCSAs of 1 cm^2^, which is rather at the higher end of the range of measured values for MF fascicles (Bayoglu et al., 2017a).

### 3.3 Vertebral Loading

**FIGURE 9:**
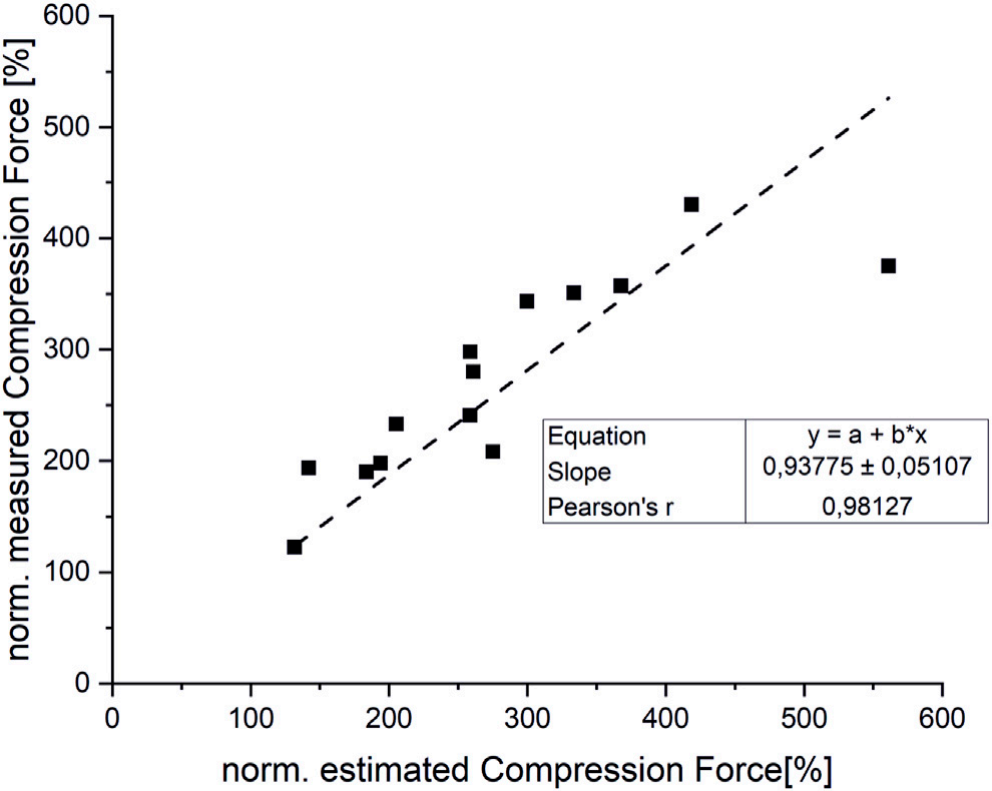
Correlation between measured and estimated compression forces (normalized to upright standing).

**FIGURE 10:**
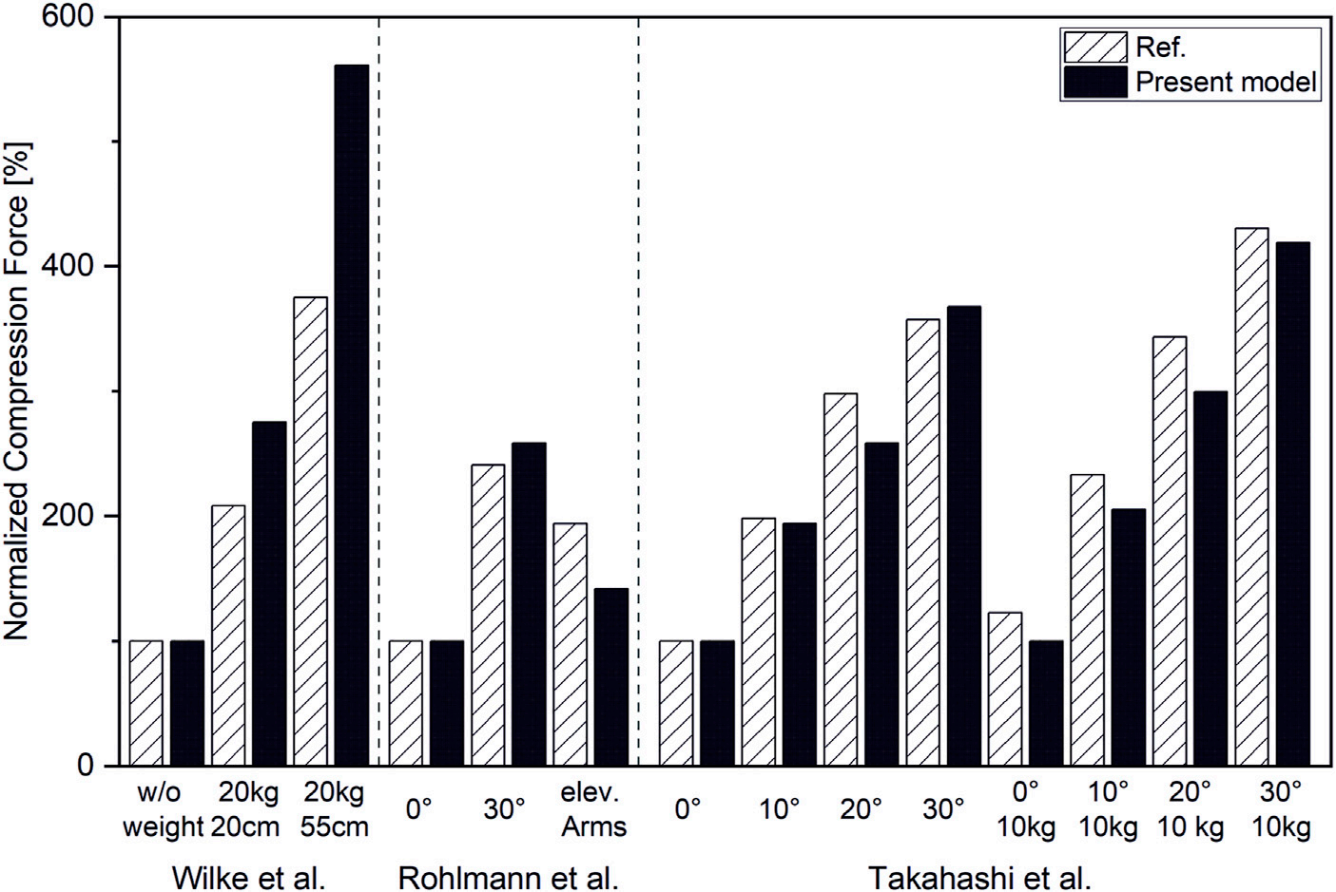
Compression forces normalized to respective forces in upright standing position for L4/L5 (Wilke et al., 2001; Takahashi et al., 2006) and T12/L1 (Rohlmann et al., 2008).

Overall, the estimated compression loads on intervertebral joints for various static loading tasks showed a good correlation with reported spinal load measurements (r = 0,98) (Figure 9). However, our model slightly underpredicted compression forces normalized to upright standing compared to measured forces from the study cited herein (Takahashi et al., 2006) by up to 16%. Normalized compression forces for upright standing with 20 kg weight held 55 cm from the Sacrum were overestimated by 33% compared to data from the respective comparative study (Wilke et al., 2001) (Figure 10).

### 3.4 Influence of Individual Characteristics on Spinal Loads

**FIGURE 11:**
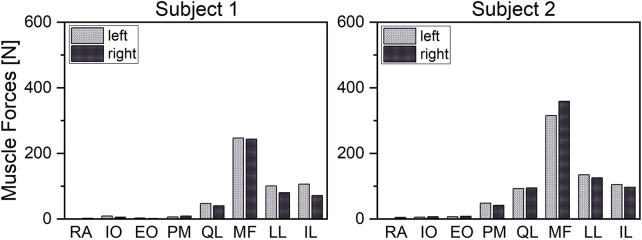
Comparison of interindividual muscle forces at 20°flexion.

**FIGURE 12:**
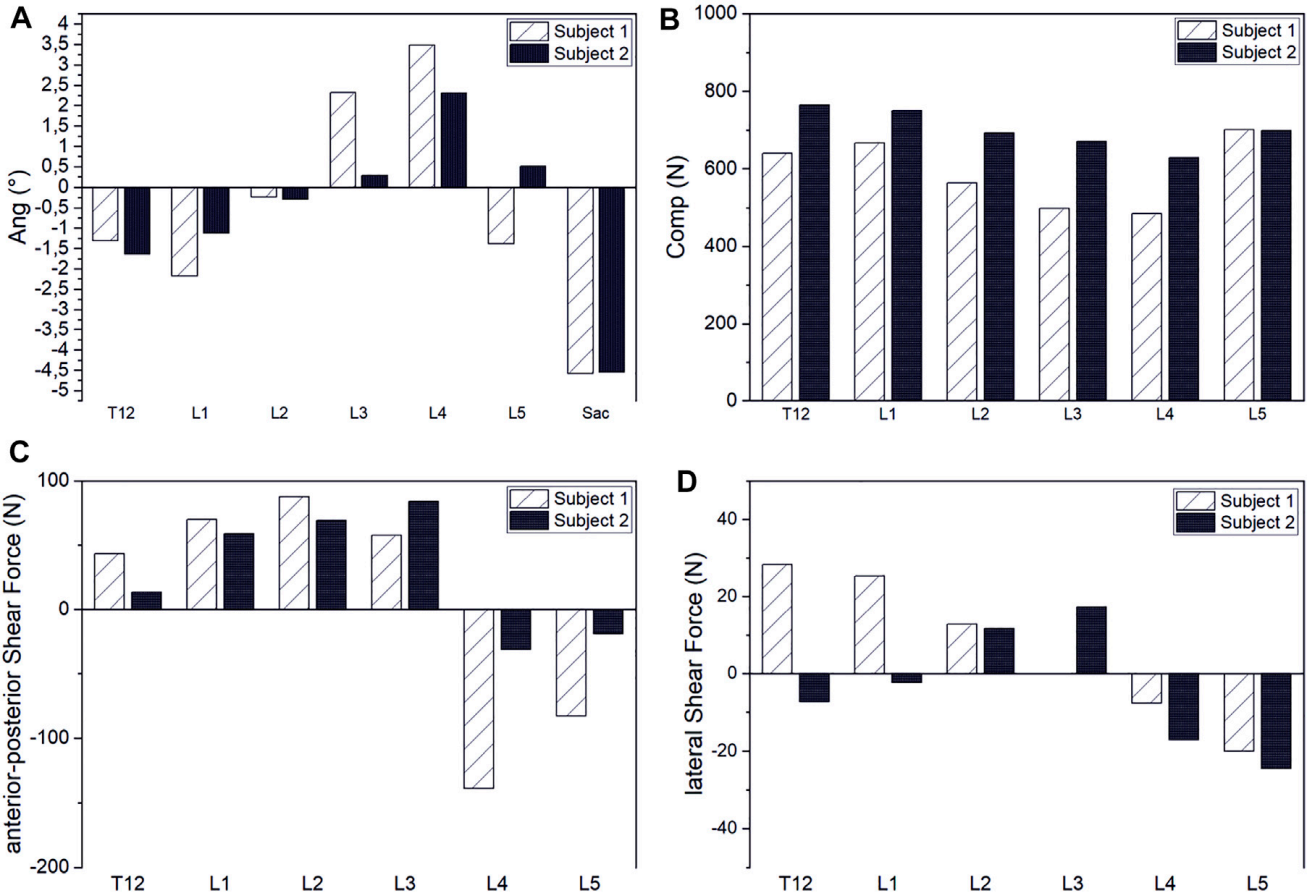
Interindividual comparison of compensation angles for upright standing **(A)** and intervertebral joint compression **(B)**, anterior–posterior shear **(C)** and lateral shear forces **(D)**.

Our results demonstrated that our patient-specific models are well suited to investigate interindividual differences. In correlation with individual spinal characteristics, distinct differences in muscle activity and vertebral loading between investigated subjects could be identified. Thus, subject two showed generally higher muscle activity than subject one (Figure 11). The compensation angles for the upright standing position were in the same range. In both cases, the balancing movement started to a large extent from a sacral rotation. However, subject one showed a rather extensive compensation in the lower mid-region of the lumbar spine, whereas subject two compensated within a smaller range in the upper lumbar region (Figure 12). Subject one showed a smaller overall compensatory flexion with -3,9° than subject two (-4,5°). The estimated shear forces showed considerable differences, especially regarding anterior–posterior loading of up to 123 N anterior and 84 N posterior for subjects one and two, respectively. Please note that this relates well to the differences in lumbar lordosis between both subjects. In terms of lateral shear forces, the differences in the thoracolumbar transition are particularly noticeable and considerably higher for the subject with thoracic scoliosis.

## 4 Discussion

We created the first pipeline for the generation of patient-specific musculoskeletal models of the spine based on CT data. The models include individual vertebrae with muscle and ligament attachment points, spinal alignment, torso weight and distribution, as well as spinal ligaments and back muscles. The models are capable of simulating static activities and estimating lumbar loads and muscle forces via a static optimization approach. The automated nature of our unique process makes it suitable for large-scale interindividual comparative studies. Thus, it holds the potential to identify biomechanical risk factors for degenerative spine diseases on a quantitative basis in larger cohorts.

The SSL contributes little to spinal stability, which is consistent with observations from the literature. This can be attributed to the fact that the SSL is the only spinal ligament featuring a negative pre-strain for the upright position (Robertson et al., 2013). The large forces occurring in the LF even with reduced pre-strain might be due to the fact that our centers of rotation are located rigidly in the center of the IVD. This is only an approximation, since the physiological instantaneous center of rotation migrates considerably posterior during reclination movement (Liu et al., 2016; Aiyangar et al., 2017). Shifting the center of rotation posterior is expected to lead to a reduced strain in the ligament and therefore to lower loading (Han et al., 2013). The modeling of intervertebral joints as fixed spherical joints is one major limitation of our model, which is expected to influence not only the ligament forces but intervertebral loads as well. To address this limitation, the model has to be equipped with additional degrees of freedom. To counteract the decreased stability associated with this, facet joints and intra-abdominal pressure should be included. This will increase model complexity and require a different optimization approach to solve the redundancy problem. Possible approaches include inverse kinematics and trajectory tracking such as computed muscle control (Liu et al., 2008; Hamner et al., 2010) or forward static optimization (Shourijeh et al., 2017). Moreover, the assumption of an average fixed lombopelvic rhythm is a further simplification, which has to be adapted, especially for larger flexion angles (Tafazzol et al., 2014).

Our model was able to predict muscle forces in close correlation with myoelectric activity measurements from the literature (Takahashi et al., 2006). To ensure satisfying equilibrium conditions for all models and load cases, we overestimated MMS and partly muscle PCSAs (IS and MF attached to the thoracic spine). However, results for maximum and mean muscle activation indicate that our model would be able to meet equilibrium conditions for lower and therefore more physiological parameters as well. Nevertheless, integration of individualized muscle architecture is desirable (Bruno et al., 2017). Past studies show that apart from individual PCSAs, the proton density fat fraction has to be considered when it comes to the estimation of maximum muscle strength (Schlaeger et al., 2019). Due to software limitations, we modeled muscle fascicles as simple point-to-point actuators. Thus, we are not able to consider paraspinal redirection of the muscle fascicles. For larger flexion angles, this leads to unrealistic lines of action and therefore, incorrect moment arms. In consequence, it is likely that muscles are over- or underactivated, depending on the mechanical state of the load case.

We predicted vertebral loading in close agreement with measured *in vivo* data, although there are discrepancies. For lifting tasks, the models tended to overestimate vertebral loading with a maximum of 33%, whereas flexion rather led to underestimation with a maximum of 16%. The reasons for those deviations can be manifold. Thus, precise flexion angles are not given in all studies (Wilke et al., 2001; Rohlmann et al., 2008). We therefore based our study design on given flexion angles from the study cited herein (Bruno et al., 2015). Regarding *in vivo* data from Wilke et al., our model tended to overestimate compression forces. This difference could possibly be explained by the fact that realigning the spine to compensate for the anterior weight is likely in order to reduce necessary muscle activity. We used an optimization approach to consider those compensation effects for an unloaded upright standing position, not however, for high-intensity lifting tasks. Yet, it is precisely in these postures, like carrying an additional 20 kg in front of the chest, in which such effects are likely to occur (Kimura et al., 2001). Thus, an external load applied at the front would lead to a balancing posture that would shift the body’s center of gravity backward and would reduce the occurring moment. Neglecting this “leaning backward” phenomenon will lead to an overestimation of muscle forces. Another possible explanation for the overestimation of lumbar loads during lifting tasks is the neglect of the stabilizing effect of intraabdominal pressure (IAD) (Hodges et al., 2005; Stokes et al., 2010). According to the study cited herein (Arjmand and Shirazi-Adl, 2006), consideration of IAD decreases vertebral load by a mean value of 19% for static loading tasks. Compared to experimental data from Takahashi et al. (Takahashi et al., 2006), our model slightly underpredicted vertebral loading. One possible reason for that is that the static optimization approach we use does not account for co-contraction (Ezati et al., 2019). Therefore, muscle activation of agonists does not have to react to forces from antagonists, which reduces overall muscle forces and, consequently, resulting joint forces. Apart from that, influencing factors due to respective *in vivo* study designs have to be noted. While Wilke et al. measured IDP via pressure sensors inserted directly into the intervertebral disc, Rohlmann et al. measured T12/L1 compression forces via a telemetrized instrumented implant (Rohlmann et al., 2008). To stabilize the spine, bisegmental spinal fixators were implanted additionally to the instrumented implants. Firstly, those fixators can lead to a relief of the measuring device and furthermore engage in natural spinal kinematics.

In our study, interindividual analysis of two subjects showed plausible results under consideration of individual characteristics. Thus, higher muscle activity and resulting vertebral loading were calculated for subject one, which can be attributed to the higher bodyweight and match findings from previous studies (Ghezelbash et al., 2016; Akhavanfar et al., 2018). Anterior–posterior shear forces were markedly increased for subject one, especially in the lower lumbar region. This can be explained by the pronounced lumbar lordosis and thus, strongly tilted vertebrae. Similar effects can be observed regarding lateral shear forces, which were more pronounced in scoliotic subject one as well. Focusing on occurring forces in the region of the thoracolumbal transition, these findings match the subject’s mild scoliosis in the thoracic spine. However, we emphasize that due to the small patient cohort, this study’s investigation of interindividual differences should be interpreted solely as a proof of concept. Further studies including larger patient cohorts are necessary to comprehensively evaluate the potential of our process for interindividual analysis.

In conclusion, we established a pipeline for automated segmentation and generation of subject-specific multibody models of the lumbar spine. We validated our biomechanical model by demonstrating a close accordance with our results with previous *in vivo* studies. Our unique approach of automatically extracting vertebral geometries including attachment points for muscles and ligaments, spinal alignment, and weight and soft tissue distribution of the trunk gives us the opportunity to systematically investigate biomechanical factors influencing spinal loading. The automation allows the analysis of large patient cohorts to gain meaningful insights into the healthy and pathological biomechanics of the spine.

## Data Availability

The raw data supporting the conclusion of this article will be made available by the authors, without undue reservation.
